# Mitochondrial Pathway of α-Tocopheryl Succinate-Induced Apoptosis in Human Epidermoid Carcinoma A431 Cells

**Published:** 2012

**Authors:** M.A. Savitskaya, M.S. Vildanova, O.P. Kisurina-Evgenieva, E.A. Smirnova, G.E. Onischenko

**Affiliations:** Biological Faculty, Lomonosov Moscow State University, Leninskie gory, 1/12, Moscow, Russia, 119991

**Keywords:** α-tocopheryl succinate, apoptosis, mitochondrial pathway, ROS, cytochrome*c*

## Abstract

Vitamin E derivatives are known to act as agents exhibiting cytotoxity against tumor cells. The effect of vitamin E succinate on human epidermoid carcinoma cell line A431 was investigated in this study using live imaging, immunocytochemistry, and transmission electron microscopy. α-Tocopheryl succinate-induced apoptotic cell death in A431 cells was shown to be both dose- and time-dependent. The hyperproduction of reactive oxygen species, changes in size, shape and ultrastructural characteristics of mitochondria followed by the release of cytochrome*c*from mitochondria to cytosol were observed. These results suggest that α-tocopheryl succinate induces apoptosis that occurs via the mitochondrial pathway. Mitochondria are shown to be crucial targets in α-tocopheryl succinate-induced caspase-dependent cell death in human carcinoma A431 cells.

## INTRODUCTION

Many researchers are now focusing on searching for anti-tumor agents that would selectively affect malignant cells while being nontoxic to normal cells and tissues. Vitamin E derivatives are among such compounds.

The term “vitamin E” is now used to refer to a large group of both the natural and synthetic compounds known as tocopherols and tocotrienols, as well as to the acetyl and succinyl derivatives of tocopherol. The biological effects of vitamin E are diverse and remain understudied. Certain forms of vitamin E can be considered as potential anti-tumor agents, since they can scavenge free radicals, suppress growth and induce differentiation in transformed cells, affect the course of the cell cycle, induce apoptosis, and activate the immune system [1–3].

It has been shown that α-tocopherol exhibits virtually no anti-tumor activity, whereas a number of its derivatives, including vitamin E succinate (α-tocopheryl succinate, α-TS), exhibit antitumor properties. Unhydrolized α-TS is a redox-silent compound ; however, as opposed to free α-tocopherol, it exhibits unique antiproliferative and proapoptotic properties [4]. α-TS can affect tumor cells in culture [5, 6], as well as human tumor xenografts in animal models and tumors induced by chemical cancerogenes [7–11]. α-TS can induce cell death and cell cycle arrest [12, 13], inhibit angiogenesis [14], and protect the organism against ionizing radiation [15].

Intracellular targets of α-TS in cell lines of different origins have been subjected to intense investigation in recent years. α-TS has been demonstrated to induce the apoptotic death of a number of tumor cells (breast cancer, malignant mesothelioma, neuroblastoma cells) via the mitochondrial pathway [16–21]. Nevertheless, the mechanisms of α-TS -induced apoptosis remain rather poorly understood. The effect of α-TS has been investigated on tumor cell lines of different origins. Skin neoplastic diseases are characterized by high malignancy and belong to poorly treatable tumors; however, there are almost no studies devoted to the effect of vitamin E derivatives on transformed keratinocytes thus far. Malignant skin diseases often have an unfavorable prognosis.

Hence, the effect of α-tocopheryl succinate on human epidermoid carcinoma A431 cells was investigated in this study.

## EXPERIMENTAL

**Cell culture and experimental procedure**

A431 cells (human epidermoid carcinoma) (Institute of Cytology of the Russian Academy of Sciences, Russia) were cultured in a DMEM medium (PanEco, Russia) supplemented with 10% fetal bovine serum (PAA Laboratories, Austria) and 80 mg/ml gentamycin (PanEco) at 37°C with 5% CO _2_ .

96% ethanol (Sigma) was used as a vehicle for α-TS. Cells cultured in the unsupplemented medium were used as the first control. Cells cultured in the medium with vehicle were the second control. Cells were treated with the agent and ethanol used as the second control on day 2 following cell seeding and incubated for 24, 48, and 72 h.

**Assessment of the level of cell death**

**Fig. 1:**
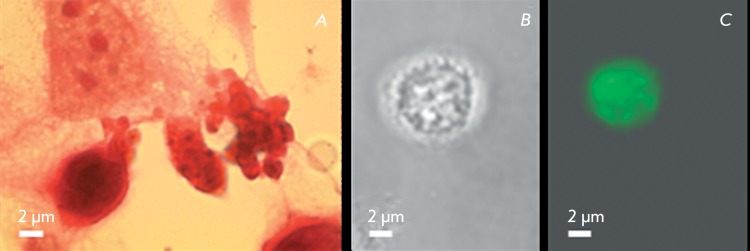
Apoptosis in human epidermoid carcinoma A431cells: a – on hematoxilin and eosin-stained slides, b – phase contrast, c – immunocytochemical revealing of caspase-3 with anti-caspase-3 antibodies in the cell shown in “b”.

The percentage of apoptotic cells in the population was counted on specimens stained with hematoxylin and eosin according to the standard procedure. Such morphological indicators as chromatin condensation, as well as cytoplasmic shrinkage and blabbing, were used as the criteria to identify the cells that went into apoptosis. The effect of α-TS at concentrations of 20, 40, 60, and 100 µM was assessed. The specimens were analyzed on a Leica DM 1000 microscope with a N PLAN 100x/1.25 Oil objective. The results were processed using the Microsoft Office Excel 2007 software.

**Cytochemistry and immunocytochemistry**

The cells for the immunocytochemical studies were fixed with 4% formaldehyde (MP Biochemicals, France) prepared using a 0.1 M PBS buffer (Sigma), pH 7.2. The specimens were stained with monoclonal antibodies against the active form of caspase-3 (Sigma) and with anti-cytochrom *c * sheep antibodies (Sigma). Anti-mouse IgG antibodies conjugated to Alexa Fluor-488 (Sigma) and anti-sheep IgG antibodies conjugated to Alexa Fluor-488 (Invitrogen, USA), respectively, were used as secondary antibodies. Cell nuclei were stained with DAPI (100 nM, Sigma). The specimens were embedded in a 1:1 PBS–glycerol solution and analyzed on an Axiovert 200M luminescent microscope (Carl Zeiss Inc., Germany) using a Plan-NEOFLUAR 100x/1.30 objective. The images were processed using the Adobe Photoshop and ImageJ software.

Mitochondria were stained with the potential-dependent dye Mitotracker Orange CMTMRos (100 nM, Invitrogen Molecular Probes). Cells were fixed with 4% formaldehyde (MP Biochemicals, France) prepared in a 0.1 M PBS buffer, pH 7.2, and embedded in a 1:1 PBS–glycerol mixture.

**Live imaging**

In order to detect ROS, the medium was supplemented with 10 µM 2’,4’-dichlorofluorescein diacetate (DCFH-DA, BioChemika, USA) for 20 min. An oxidized fluorescent product, dichlorofluorescein, is formed in the presence of ROS (oxygen peroxide, peroxide anion, peroxide radical). DCFH-DA was added 48 h after α-TS had been added into the culture medium. Imaging was performed on an Axiovert 200M luminescent microscope (Carl Zeiss Inc.) using a Plan-NEOFLUAR 20x/0.50 objective. The percentage of ROS-positive cells in the images was counted. The data were statistically processed using the Microsoft Excel software. The images were processed using the Adobe Photoshop CS3 software.

**Transmission electron microscopy**

Cells were fixed with 2.5% glutaraldehyde (Sigma) with 2% formalin in 0.1 M PBS, pH 7.2 (Sigma) and postfixed with a 1% OsO _4_ solution (Sigma) in PBS for 1 h in dark conditions. The standard procedures for transmission electron microscopy were then used. Ultrathin sections (60–80 nm) were cut with an Ultratom-5 ultramicrotome (LKB, Sweden). The sections were contrasted with a 1.5% aqueous solution of uranyl acetate (Serva, USA) and Reynolds’ lead citrate (Serva). The specimens were examined in a JEM-1011 electron microscope (JEOL) equipped with a GATAN ES500W digital camera with the Digital Micrograph software (GATAN) and a JEM-100B transmission electron microscope (JEOL).

## RESULTS AND DISCUSSION

**α-TS induces apoptotic death of A431 cells in time- and dose-depended manner **

The only type of cell death (apoptosis) was detected in specimens stained with hematoxylin and eosin. Apoptotic cells could be identified by using a number of criteria, such as cytoplasmic shrinkage, acquiring a round shape, chromatin condensation, blabbing, and disintegration into apoptotic bodies. No necrotic cells were detected ( *Fig. 1A* ). The presence of the active form of caspase-3 ( *Fig. 1B,C* ) confirms an apoptotic pathway of cell death.

**Fig. 2:**
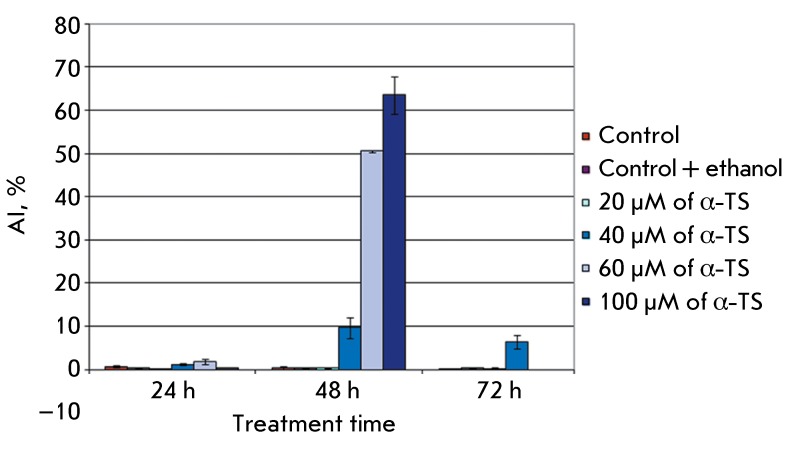
α-TS induces apoptosis in A431 cells in a dose- and time-depending manner.

**Fig. 3:**
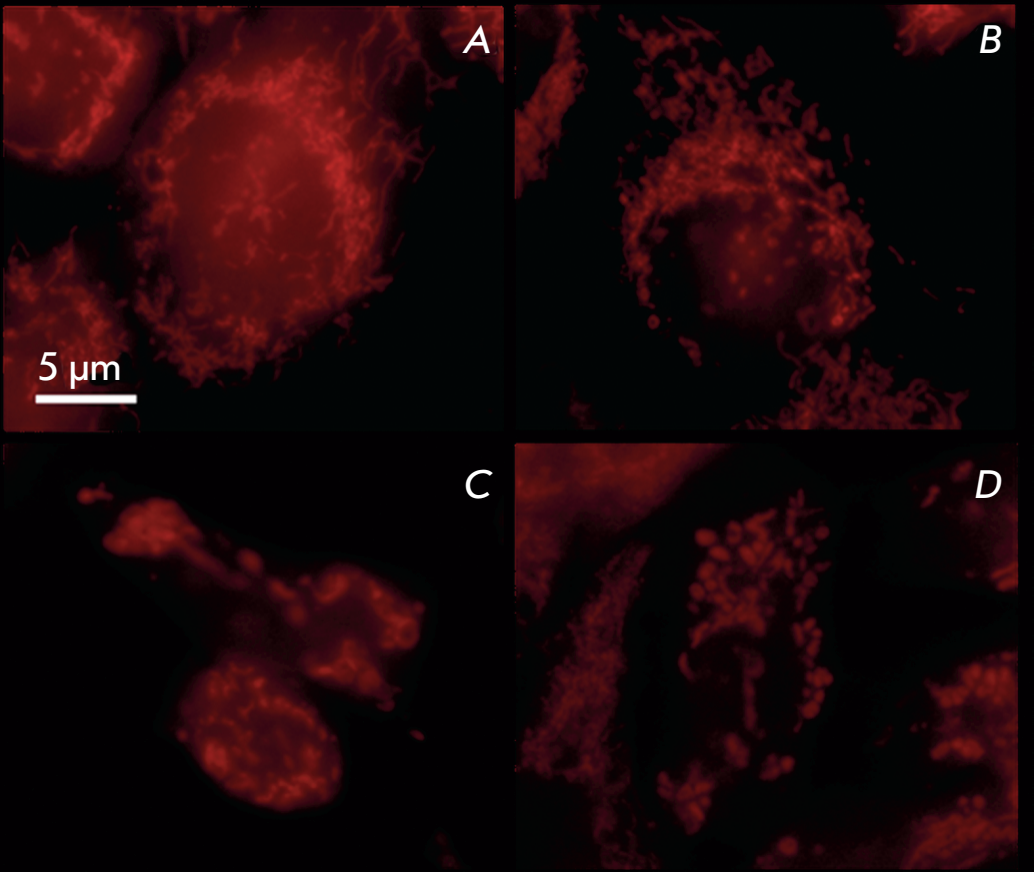
Mitochondria in A431 cells stained with MitoTracker Orange: a – control; b – control with vehicle; c, d – 40 µM α-TS, 48 h.

The apoptotic index (AI) value in the control specimens of the A431 cell culture was 0.4–0.9%; the supplementation with 96% ethanol had virtually no effect on the AI value. AI increases significantly (9.67%) on day 2 of incubation of the cells with α-TS at a concentration of 40 µM; on day 3, it remains at this level. Treating the cells with 60 µM α-TS on day 1 virtually does not alter the AI; on day 2, it increases abruptly (by over 60%), and on day 3 no cells are detected on glass slides. A similar result was obtained upon the addition of 100 µM α-TS (63.5%); however, the AI values were the highest in this case ( *Fig. 2* ).

Thus, the statistical analysis demonstrated that treatment with α-TS results in an increase in the level of A431 cell death in a time- and dose-dependent manner.

40 µM α-TS after incubation during 48 h causes a significant increase in the AI value; however, mass cell death has yet to occur. This dose was, therefore, selected for the investigation of the mechanism of apoptosis induction.

**α-TS alters the mitochondrial structure and induces the release of cytochrome **

*c* **from mitochondria into the cytosol**

According to the available data, α-TS triggers apoptosis in a number of cell lines via the mitochondrial pathway. To understand the role of the mitochondrial mechanism in apoptosis induction, the general structure of chondriome, the mitochondrial ultrastructure, localization of cytochrome *c* , and the level of ROS were analyzed. .

In order to analyze the state of chondriome in control and treated cells , the cells were stained with the potential-dependent dye Mitotracker Orange CMTMRos, which accumulates only in functional mitochondria. *Fig. 3A,B* shows A431 cells in which the chondriome is formed by numerous mitochondria (small oval, round-shaped, filamentary, curved mitochondria, etc.). Mitochondria are typically uniformly distributed over the cytoplasm; they cluster around the nucleus very rarely and sometimes are localized in the peripheral cytoplasm. Large oval and round-shaped mitochondria occur in certain cases ( *Fig. 3A* ). Ethanol treatment does not alter the distribution and shape of mitochondria ( *Fig. 3B* ).

α-TS treatment of A431 cells results in changes in the shape of mitochondria; numerous large round-shaped and oval mitochondria that are significantly larger than those in the control cells emerge. The number of these mitochondria per cell may vary; however, in general, their number is usually noticeably smaller than that in the control cells. Localization of cytochrome *c* was determined immunocytochemically. The release of cytochrome *c* from mitochondria into the cytosol is the crucial stage in the apoptosis occurring via the mitochondrial (“internal”) pathway.

**Fig. 4:**
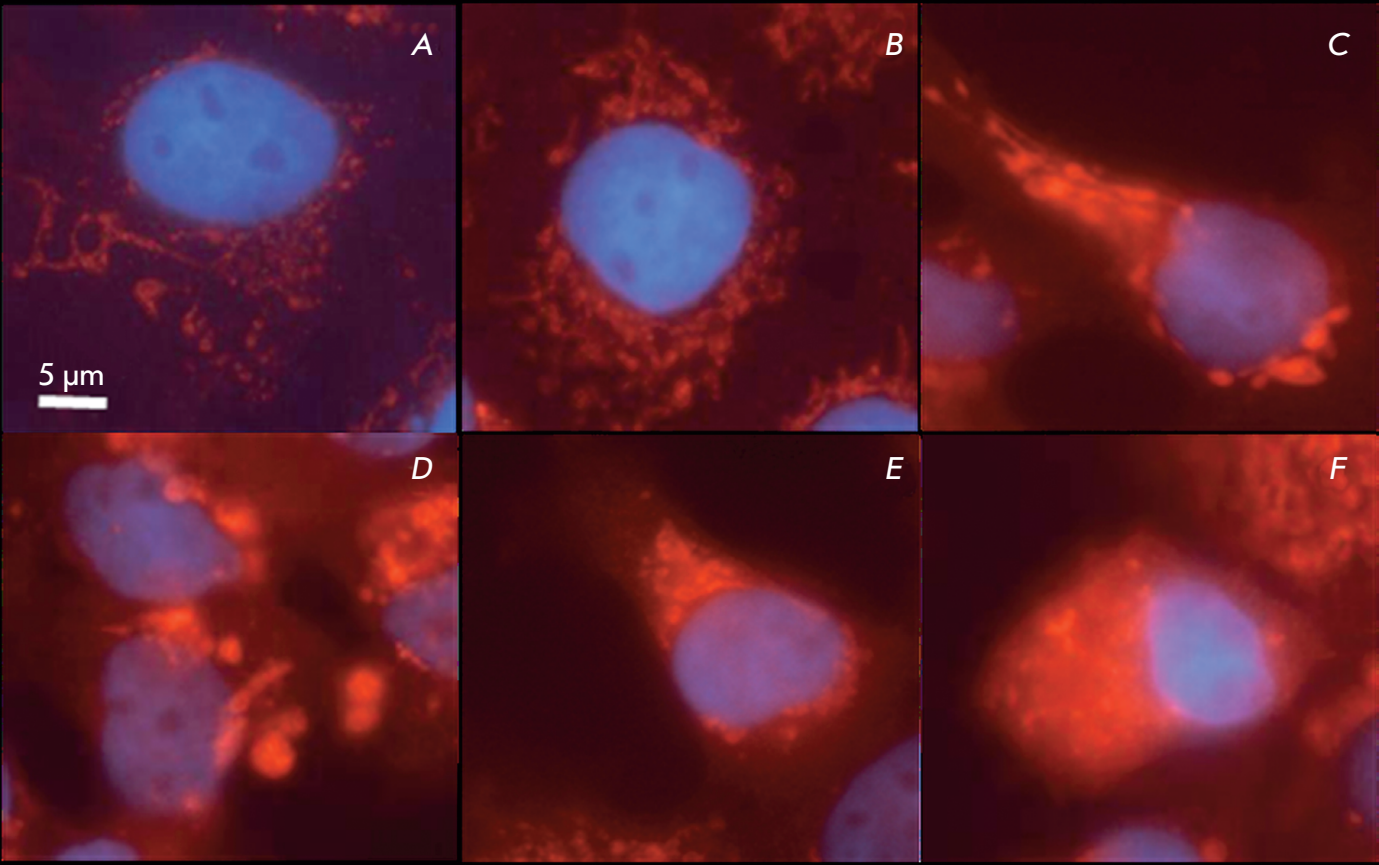
Immunocytochemical staining of A431 cells with anti-cytochrome *c* antibodies: a, b – contol; c–f – 40 µM α-TS, 48 h.

In the control and control with vehicle, anti-cytochrome *c* antibodies detect this protein within mitochondria. It is clear from *Fig. 4A,B* that there are numerous small mitochondria that are frequently filamentary shaped. Small oval and round-shaped mitochondria occur in some cases.

Different degrees of cytoplasmic staining and different amounts of stained mitochondria can be traced in cells cultured in the presence of α-TS ( *Fig. 4C–F* ), which attests to the fact that cytochrome *c* is released from some mitochondria into the cytosol. Thus, *Fig. 4D * shows a cell with mitochondria of increased size and round and oval shapes. Meanwhile, the cytoplasm remains virtually unstained. *Fig. 4D* shows a cell with mitochondria of a size similar to that of mitochondria in the control cells. However, unlike them, mitochondria shown in *Fig. 4D * are short oval bodies. The release of cytochrome *c* from mitochondria into the cytosol can be seen in *Fig. 4F* . It should be mentioned that some mitochondria contain cytochrome *c* .

**Fig. 5:**
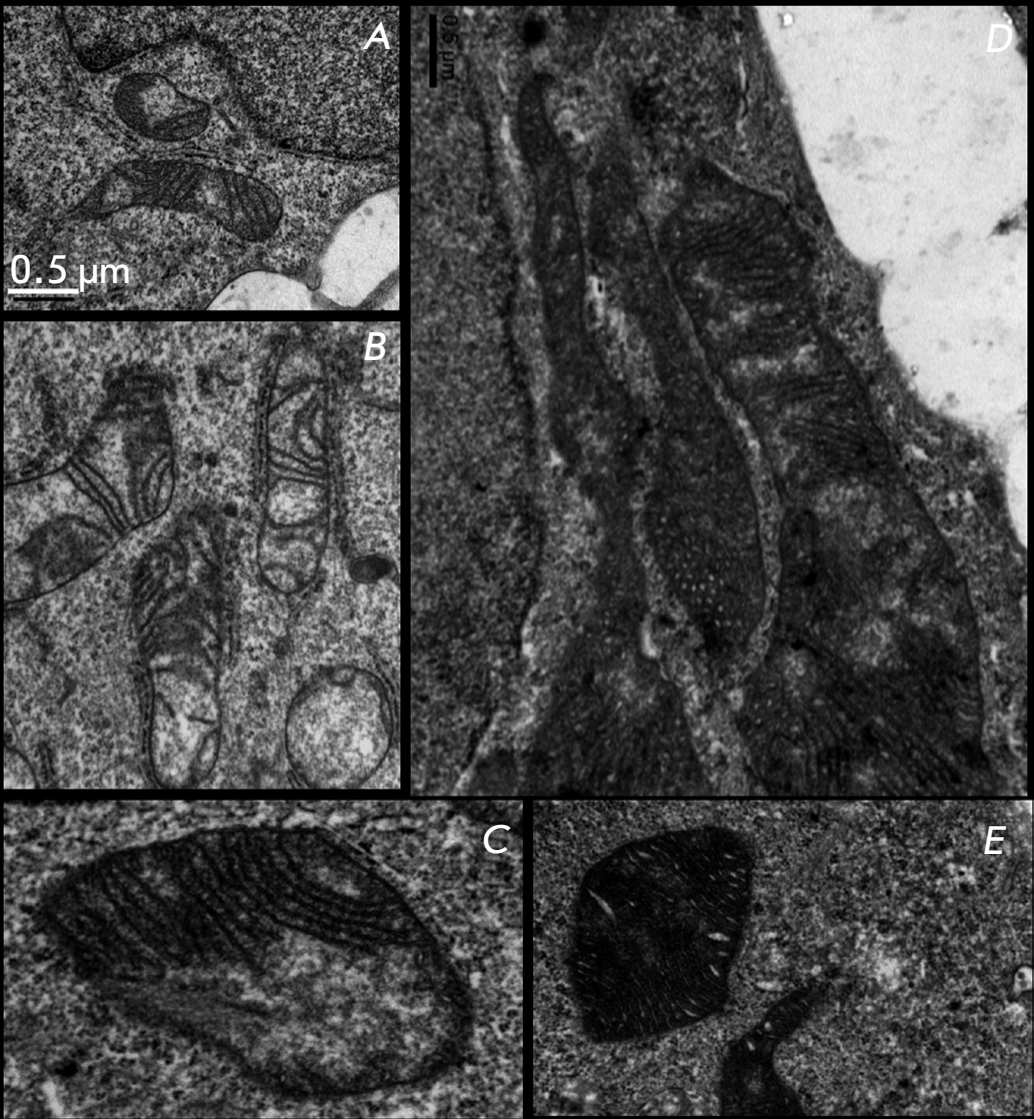
Ultrastructure of mitochondria in A431 cells:  a – control; b – control with vehicle; c–e – 40 µM α-TS, 48 h.

An ultrastructural investigation of control A431 cells revealed small mitochondria with a light matrix and relatively sparse thin cristae ( *Fig. 5A,B* ). Treatment with α-TS results in significant alterations in mitochondria ( *Fig. 5C–E* ). Sections may contain giant mitochondria with a large number of cristae or mitochondria with invaginations, certain areas of which are filled with numerous cristae ( *Fig. 5D). * Certain mitochondria contain a dense matrix and dilated cristae ( *Fig. 5E* ). There are also mitochondria with an ultrastructure virtually identical to that of mitochondria in the control cells. It is interesting to note that mitochondria with different ultrastructures can occur in the cytoplasm of one cell. The heterogeneity of the mitochondrial population presumably represents different stages of alterations occurring under the action of α-TS.

**α-TS increases the ROS level in A431 cells**

**Fig. 6:**
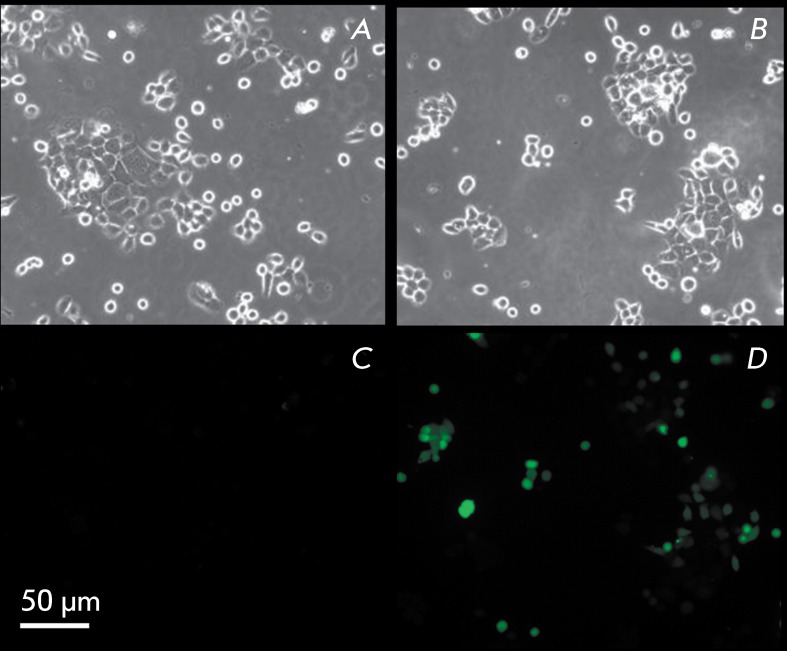
Live-imaging of A431 cells stained with DCFH-DA:a, с – control; b, d – 40 μM α-TS.

**Fig. 7:**
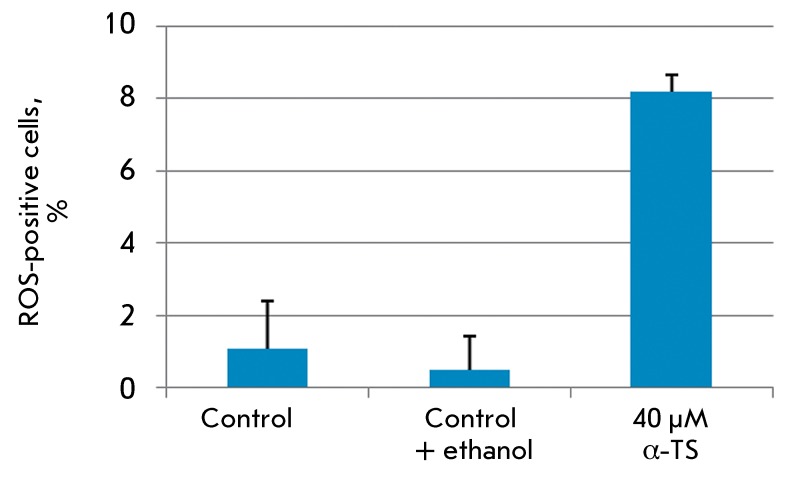
Live-imaging of A431 cells stained with DCFH-DA: percentage of peroxide-positive cells in the control and after the incubation with 40 µM α-TS.

Cell death can be mediated by an increase in ROS production. In this regard, live imaging of cells with DCFH-DA enables one to detect hydrogen peroxide. Individual fluorescent cells ( *Fig. 6A,B* ) were detected in the control; ethanol caused no noticeable changes.

Treatment with 40 µM α-TS for 48 h significantly enhances ROS production in cells. The percentages of stained cells ( *Fig. 7* ) are relatively small both in the control and vehicle-treated cells (0.08 and 0.49%, respectively). Meanwhile, treatment with α-TS considerably increases the percentage of cells with excessive content of ROS (8.18%).

Thus, this study demonstrates that α-TS induces apoptotic cell death in human epidermoid carcinoma cell line A431 in a dose-dependent manner. In morphological terms, morphological characteristics of apoptosis are revealed, such as blabbing, chromatin condensation, nuclear fragmentation, and disintegration of a cell into apoptotic bodies. Furthermore, the apoptotic pathway of cell death was also confirmed by the fact that the cells were stained with antibodies against the active form of caspase-3. No cells with necrotic appearance, such as cell swelling, were observed. α-TS has been reported to induce apoptotic death of the following cell lines: gastric [22], colon [10], breast [23, 16], prostate [17], lung [24], cervical, ovary [25] cancer cells; hepatoma cells [26], osteosarcoma cells [12, 13], Jurkat T cell lymphoma cell line [8, 18, 29], and the other malignant hematopoietic cell lines [8, 19]; murine melanoma and glioma cell lines, and rat and human neuroblastoma [30, 31]. Apoptosis has been shown to be induced by α-TS in micromolar concentrations; the effect is time- and dose-dependent [8, 18, 32–35]. We have demonstrated that apoptosis induction in human epidermoid carcinoma cells is also time- and dose-dependent. Unlike α-tocopherol, which is known for its antioxidative properties, α-TS is a redox-silent compound and does not exhibit antioxidant properties [35]. Contrariwise, we and other authors [21] have shown that α-TS can act as a pro-oxidant in tumor cells and enhance generation of oxygen radicals.

It has been suggested in a large number of studies that mitochondria are the major targets in α-TS action on tumor cells [18, 19, 35, 36–38]. We have revealed a significant change in the mitochondrial ultrastructure and release of cytochrome *c* from mitochondria into the cytosol of A431 cells. We can assume that enhanced production of ROS and release of cytochrome *c * are related processes. Abundant data confirm the fact that α-TS can considerably enhance ROS generation in various cell lines, such as human and mouse breast tumor cells, a Jurkat T cell lymphoma cell line, Chinese hamster lung fibroblast cells, malignant mesothelioma cells, and human head and neck carcinoma cells [27, 36–40]. It has been mentioned in most studies that the superoxide anion radical plays a central role in apoptosis, which follows hyperproduction of ROS; however, Gu *et al* . [40] have demonstrated that hydrogen peroxide is dominant in epidermoid carcinoma cells, whereas the amount of superoxide is negligible. A significant increase in the percentage of cells exhibiting hyperproduction of ROS has also been revealed in this study. A DCFH-DA dye can interact with hydrogen peroxide; therefore, we have revealed the formation of hydrogen peroxide. Since peroxide is generated in cells from a superoxide anion radical, it is most likely that O _2_^-^ acts as the primary form of oxygen radicals.

Within the cell, the major source of ROS formation is mitochondria, where free radicals are generated due to the function of the electron transfer chain. α-TS has been shown to be capable of inhibiting the activity of complexes I [41] and II of the mitochondrial respiratory chain. The inhibition of complex II by α-TS has been observed in breast cancer cells, Jurkat cells, and rat thymocytes [36, 42, 43]. The activity of complex II has been reported to decrease due to the fact that α-TS acts as a pseudosubstrate for succinate dehydrogenase by binding to the Q _P_ and Q _D _ sites of the enzyme complex. Thus, the inhibition is competitive. When ubiquinone is replaced with α-TS , the electrons in the binding site of ubiquinone are not transported to FAD, [Fe-S] sites, haem, and ubiquinone via the hydrophilic part of succinate dehydrogenase. Instead, they recombine with molecular oxygen, yielding a superoxide anion radical; its accumulation may eventually result in the apoptosis of tumor cells [38].

It is a known fact that enhanced generation of ROS may trigger apoptosis through the mitochondrial pathway. ROS can mediate the formation of disulfide bonds between the Bax monomers in the cytosol, which results in the formation of channels in the outer mitochondrial membrane [42] and disturbs the binding of cytochrome *c* to cardiolipin, the mitochondrial membrane phospholipid, thus causing its hydroperoxidation [43, 44]. ROS generated in α-TS-treated cells can induce cytochrome *c* dissociation from cardiolipin and release of the protein into the cytosol, where cytochrome *c* induces caspase activation.

It is remarkable that the release of cytochrome *c* from all mitochondria is not a simultaneous process. Several mitochondria containing cytochrome *c * can be retained even in those cells where cytoplasm has been considerably strongly stained with anti-cytochrome *c* antibodies. These mitochondria are usually of increased size and oval or rounded shape. Large mitochondria are also detected by staining cells with a Mitotracker Orange potential-dependent dye. Since the release of cytochrome *c* requires disturbing the permeability of the mitochondrial membrane, the release must be accompanied by a decrease in the mitochondrial inner membrane potential. Thus, we have demonstrated that mitochondria containing cytochrome *c* and having the membrane potential (i.e., the mitochondria participating in the synthesis of ATP, whose production is required even at the late stages of apoptosis, the energy-dependent process) can be retained in cells upon treatment with α-TS .

## CONCLUSIONS

We have demonstrated that mitochondria are the crucial target of α-TS action in epidermoid carcinoma A431 cells. α-TS was found to alter the shape and ultrastructure of mitochondria and enhance ROS production and release of cytochrome *c* from mitochondria into the cytosol, which induces caspase-dependent apoptosis. These findings enable us to propose the following mechanism of α-TS-induced cell death. α-Tocopheryl succinate inhibits the function of the respiratory chain complex II, which results in the disturbance of electron transport and acceleration of ROS formation. In turn, ROS accumulate in a cell and cause mitochondrial damage, which leads to the release of cytochrome *c* into the cytosol and triggers the caspase-dependent apoptotic cell death program. 

## Abbreviations

α-TS: α-tocopheryl succinate
ROS: reactive oxygen species
AI: apoptotic index
